# Khat Use Patterns, Associated Features, and Psychological Problems in a Khat-Treatment-Seeking Student Sample of Jimma University, Southwestern Ethiopia

**DOI:** 10.3389/fpubh.2021.645980

**Published:** 2021-08-19

**Authors:** Mekdem Tesfamichael Hassen, Matiwos Soboka, Marina Widmann, Lucas Keller, Anja C. Zeller, Natascha Büchele, Eva Barnewitz, Yimenu Yitayih, Sabine Schiller, Jael Senger, Kristina Adorjan, Michael Odenwald

**Affiliations:** ^1^Department of Psychology, University of Konstanz, Konstanz, Germany; ^2^Department of Psychiatry, Medical Faculty, Jimma University, Jimma, Ethiopia; ^3^Vivo International e.V., Konstanz, Germany; ^4^Department of Psychiatry and Psychotherapy, University Hospital, Ludwig-Maximilians-Universität Munich, Munich, Germany; ^5^Institute of Psychiatric Phenomics and Genomics, University Hospital, Ludwig-Maximilians-Universität Munich, Munich, Germany; ^6^Center for International Health (CIH^LMU^), Ludwig-Maximilians-Universität Munich, Munich, Germany

**Keywords:** khat, mental health, alcohol, university students, Ethiopia, brief intervention, common mental disorder, posttraumatic stress disorder

## Abstract

**Background:** Khat (*Catha edulis*) is a traditionally used substance in African and Arab countries that contains the amphetamine-like alkaloid cathinone. Khat use among Ethiopian students is a growing concern. This study aims to describe khat use, psychological problems, and motivation to change and to determine associated factors of khat use among students from Jimma University seeking psychological assistance.

**Methods:** In a cross-sectional study, a sample of 717 students from Jimma University, southwestern Ethiopia, who seek assistance to reduce khat use were recruited. The study used Amharic and Afaan Oromoo language versions of common psychological instruments and employed them as part of a comprehensive tablet computer-delivered self-report assessment battery, comprising the SRQ-20, the PCL-5, the LEC-5, the AUDIT, and the SOCRATES-khat. In addition, socio-demographic, economic variables, and functioning problems due to severe mental disorders were assessed. The analysis relied on the data of the 575 included participants and used clinical cut-off values to describe this treatment-seeking sample and hierarchical regression models to determine variables associated with khat use.

**Results:** The sample showed high khat use in the past month (*M* = 31.55 bundles, *SD* = 28.53, on *M* = 15.11 days, *SD* = 8.54); 17.0% showed highly problematic use. The sample was extremely burdened with comorbid psychiatric problems: 21.6% reported functioning problems due to past mental disorders, 60.2% scored above the cut-off for current common mental disorders, 37.9% screened positive for PTSD, and 47.1% reported hazardous alcohol use. Small to medium intercorrelations between variables were detected, and in hierarchical regression models, higher motivation to change khat use was associated with higher use of the substance.

**Conclusions:** This study clearly shows the need to develop research instruments, screening methods, and assistance services for khat-using students at Jimma University. Study participants' high mental health burden shows the need for targeted intervention programs that go beyond brief interventions for khat use. Furthermore, the study highlights challenges for implementing such services: the barriers to utilization for females and khat users without comorbid mental health problems.

## Introduction

The leaves of the khat tree (*Catha edulis*) are a traditional psychoactive substance; khat is deeply rooted in the cultural and religious practices of certain ethnic groups in parts of Africa and the Arab Peninsula [Ethiopia, Kenya, and Yemen; (1)]. Throughout the last century, khat developed from a niche crop to a cash crop. Its production became widespread across the whole region, and consumption spilled over to the general population (2). The leaves are typically chewed for the mildly stimulating effects, mainly during social gatherings. Fresh khat leaves contain several psychoactive alkaloids of which the main principle is cathinone, S(-)-α-aminopropiophenone (3). Cathinone acts in the central and peripheral nervous systems in a similar way to amphetamine, and humans experience its effect as euphoria, making them confident, alert, and focused (4); that is the reason why it has been called “natural amphetamine” (3). Khat use has been linked to numerous health problems (5), it is legally banned in many countries outside the khat belt (6), and its increased production in the traditional use countries is discussed in the context of environmental challenges, income generation for small farmers, and food insecurity (7). In general, there is a controversial societal debate about the substance in all traditional use countries [for a review, see (2)].

Khat use is a common practive in Ethiopia [overall 30-day prevalence rate 15.3%; (8)], especially among Ethiopian university students [23% on a national level; (9)]. Previous studies with representative student samples from Jimma University found rates of around 25% (10, 11); in a sample with high school students, a prevalence of 16% was observed (12).

The subjective positive effects on alertness and concentration increase the substance's attractiveness for high school and university students and young professionals to improve academic performance (13, 14). However, the currently available evidence from cross-sectional studies points into the direction that students' khat use is related to worse academic performance (10, 11, 15) and higher mental distress (12, 16).

Several studies found increased rates of depression, posttraumatic stress disorder (PTSD), or common mental disorders (CMD) among khat users [e.g., (17–19)]. Studies with representative samples from Jimma town revealed a prevalence of CMD among the general population of between 25.8 and 33.6% (20, 21) and among medical students of 35.2% (16); in all three studies, CMD was positively associated with khat use. A recent meta-analysis (22) found a pooled prevalence of CMD among Ethiopian students of 37.73% (95% CI: 30.43, 45.03) and a clear association to khat use (OR 2.01; 95% CI: 1.38, 2.95). Excessive khat use is related to developing an addiction syndrome [for a review, see (23)]. A study conducted in the psychiatric wards of Amanuel and St. Paul hospitals (Addis Ababa, Ethiopia) by Fekadu et al. (24) revealed that the most common substance of abuse among psychiatric patients is khat (21%). Several studies revealed that khat use in Ethiopia is associated with the use of other substances, especially alcohol [e.g., (25, 26)], and that khat users with mental distress used alcohol more often to cut down stimulant effects of khat (20). Furthermore, khat use has been associated with the occurrence of psychotic symptoms and disorders [for review, see (27)]. It is unclear whether khat users seek psychological assistance or counseling because of their khat use, as it seems that users reject assistance (5, 28). Currently, the prevalence of mental problems among khat users who look for psychological assistance is unknown.

Because khat use has been linked to mental distress, we want to expand the scope of research by studying khat use and psychological health in a sample of khat-using university students who are actively seeking psychological assistance (in the context of our study, we offered a brief intervention, that is, psychological assistance to reduce or stop khat use, that otherwise did not exist). This study aims to get further information on who is seeking psychological assistance because of khat use problems, which is helpful for planning services. In addition to describing a treatment-seeking student sample in detail, we hypothesize that the severity of khat use is related to (1) the severity of comorbid mental health problems, alcohol use, and trauma load as well as (2) to a higher level of motivation to change khat use.

## Materials and Methods

### Study Location

The study was conducted at Jimma University, which is located in the southwestern part of Ethiopia. The university was established in 1952, the number of students is currently 42,000, with the number of staff members being 2,600. It is one of the country's largest institutes of higher education. Currently, the university consists of four different campuses located in different parts of Jimma town. Jimma is located in one of the country's traditional khat growing regions with above-average use of the substance (8).

### Study Design and Sampling

Using a cross-sectional design, the study included a convenience sample of khat using Jimma University (JU) students of the second study year or higher who wanted to reduce or stop their khat use and who had a strong interest in using a free psychological assistance service support them to achieve this goal. We excluded first-year students because khat use onset is often during university education (10). This study served as a preparatory study for a randomized controlled trial and had the aim to screen and recruit participants. The recruited subjects should be considered a treatment-seeking sample.

### Recruitment, Study Procedures, and Participants

With the assistance of student committees, we distributed information leaflets in the different campuses with information about study purposes (i.e., a study on psychological assistance for khat-using students) and selection criteria (i.e. student status at JU, second study year or higher, khat use in the last month, motivation to reduce or stop khat use). Potential participants were invited to participate in a brief preparation workshop in which detailed information on the study was given, that is, that a brief intervention to stop or reduce khat use will be provided. Participants who gave informed consent were admitted to the screening assessment. The screening was a detailed self-report implemented with tablet computers using the assessment software Qualtrics (https://www.qualtrics.com). Participants did not report their names in the electronic assessment but received a code (pseudonym). After a brief individual instruction, participants worked on the tablets on their own, sitting in a classroom with up to 10 others who also completed the assessment; if needed, they received assistance from a supervising staff member who was present in the classroom but did not see the participants' data entry. Instructions, questions, and predefined answers were either presented in Amharic, Afaan Oromoo, or English; participants selected their preferred language from these possibilities. The assessment took on average 45 min. Participants were reimbursed for their public transport expenses and received a lunch voucher if they missed their university-provided lunch because of the assessment. The screening took place from November 5, 2018, to November 23, 2018.

In total, 717 subjects (708 males and 9 females, i.e., 98.7 vs. 1.3%) decided to participate in the screening (Age: *M* = 22.06, *SD* = 1.63; Study Year: *M* =3.40, *SD* = 1.08). *N* = 575 could be included in our analyses. We excluded all females to be able to interpret results clearly because, in the Ethiopian societal context, khat use among women has to be considered a distinct phenomenon to male khat use (14), and the small number of female participants did not allow for any analysis of potential gender differences. In line with our recruitment criteria, we also excluded all first-year and non-regular students (*N* = 13 and *N* = 3, respectively) that took part in the screening. In addition, we excluded 65 students because they did not report any khat use in the previous month, 15 students because they reported extreme amounts of khat use that were implausible (see the definition of extreme cases below), and 37 students because they had left out three or more items in one or more of the questionnaires (for more information see the section s*tatistical procedure* below).

Last khat use before the assessment occurred on the day of the assessment itself among 47.8% (*N* = 275; number of bundles: *M* = 2.10, *SD* = 1.73) and on the day before the assessment in 19.3% (*N* = 106; *M* =1.81, *SD* =1.40).

### Instruments

All instruments and manuals had been made available in the two widely spoken languages in the region as well as inside the campuses (Afaan Oromoo and Amharic) and in English. If validated versions of the instruments had not been available, we used a combination of the back-translation standard and the committee approach to develop the missing versions (29): To do this, instruments were first translated and then independently back-translated; additionally, an international committee of Ethiopian and German scholars discussed the properness of the translated and back-translated contents, comparing it to the original versions of the instruments and modifying it, if necessary; at last, the instruments and the conceptual correctness of their translation were again discussed during the training of local counselors who learned to apply the instruments in a standard way by role-plays and supervised field tests and adapted if problems emerged.

### Severe Mental and Neurological Disorders

We assessed severe functioning problems caused by psychiatric and neurological disorders with a series of questions that had been validated and used in previous research in the region (30): “*Have you ever been unable to work, go to school, or fulfill your household or childcare duties for at least 4 weeks because of mental or nerve-related problems?*” If the participant answered yes, the follow-up question was “*Have you been unable to work, go to school, or fulfill your household or childcare duties because of mental or nerve-related problems in the last four weeks?*”. In a next step we asked “*Have you ever sought assistance from a counselor, medical doctor, priest, sheikh, or healer because of mental or nerve-related problems?*” and “*Have you sought for such assistance in the last 4 weeks?*” We used the first question to screen for the lifetime presence of any mental disorder.

### Khat Use

The *Timeline Follow Back* (TLFB) method is a well-validated and frequently employed calendar-based self-report assessment originally developed for alcohol use but appropriate for other substances, too (31). It has been validated in cross-cultural studies (32). In previous research projects in the Jimma region, the TLFB was adapted and used to assess khat and alcohol use (33). For this study, participants used the TLFB calendar to report the consumed standard bundles of khat as well as days with khat use in the last 28 days before the assessment. Standard units of different khat qualities had been determined beforehand by a local expert committee using a market survey and consensus discussions as outlined by (34). Respondents received descriptions and photos of the different standard units. As a common definition of problematic khat use does not yet exist (14), we used the quantity of consumption as a marker for highly excessive khat consumption. In previous studies, a khat use of two or more bundles a day (35, 36) was a marker for problematic khat use. Here we used 56 or more bundles of khat in 28 days (i.e., on average, two or more bundles per day) as a marker for *highly problematic khat use*.

### Hazardous Alcohol Use

The *Alcohol Use Disorder Identification Test* (AUDIT) (37) is a brief screening instrument with 10 items developed by the WHO to identify individuals with problematic alcohol use (sample item “*How often during the last year have you failed to do what was normally expected of you because of drinking*?”). A sum score of 8 and above is recommended as the cut-off for hazardous and harmful alcohol use; scores of 8 to 15 are interpreted to represent a medium-level and scores of 16 or above a high-level alcohol problem. An Amharic version was developed by Gebrehanna et al. (13) and was tested with Ethiopian university students. We developed an Afaan Oromoo version and found good Cronbach's alpha coefficients for the three language versions of the instrument (Amharic; α = 0.77; Afaan Oromoo: α = 0.81; English: α = 0.81).

### Symptoms of Common Mental Disorders

The 20-item version of the *Self-Report Questionnaire* (SRQ-20) developed by the WHO (38) comprises 20 simple questions that need to be answered in a yes-no format (e.g., “*Do you feel nervous, tense or worried?*”). A validated Amharic version of the instrument exists (39), and an Afaan Oromoo version was developed in one of our previous studies (33). The instrument had been used to screen for CMD among JU students with the frequently used cut-off score of 7/8 (i.e., 7 or lower means negative screening, 8 or higher positive screening), identifying a proportion of 35.2% (16). The Cronbach's alphas for the three language versions of the SRQ-20 in our study were good to excellent (Amharic: α = 0.90; Afaan Oromoo: α = 0.90; English: α = 0.71).

### Traumatic Experiences

To assess traumatic experiences, we used the *Life-Event Checklist for DSM-5*, a well-validated instrument that assesses 17 types of potentially traumatic experiences (40), for example, “*Physical assault (for example, being attacked, hit, slapped, kicked, beaten up)*”. Respondents report whether each item happened to them personally, whether they witnessed that it happened to somebody else, whether they learned that it happened to a close person, or whether it is part of their job. Simplified Amharic and Afaan Oromoo versions of the instrument, that just asked in a single item whether each event type has ever happened to the respondents personally, whether they have ever witnessed it happen to somebody else, or whether this experience had been part of their job had been used to assess traumatic experiences in a previous study (33). We also used the simplified version in English. Trauma load was calculated by summing up the event types that had been experienced, witnessed, or part of the job. In our study, the Cronbach's alphas for the simplified LEC-5 in the three languages were good (Amharic: α = 0.87; Afaan Oromoo: α = 0.89; English*:* α = 0.88).

### Posttraumatic Stress Disorder

The *PTSD Checklist for DSM-5* is a well-validated self-report instrument to assess symptoms of Posttraumatic Stress Disorder that has recently been adapted to the criteria of DSM-5 (41). The instrument asks respondents to indicate and categorize the most distressing event and asks for the intensity of 21 symptoms in the last month, for example, “*Suddenly feeling or acting as if the stressful experience were actually happening again (as if you were actually back there reliving it)*”. Amharic and Afaan Oromoo versions of the instrument had been developed for this study. For screening PTSD, a cut-off score of 32/33 (i.e., 32 or lower means negative and 33 or higher positive screening outcome) is recommended (41). Cronbach's alphas of the three language versions used in our study can be considered good to excellent (α > 0.80). The Cronbach's alphas for the PCL-5 subscales in three languages were good to excellent, with coefficients ranging from 0.83 to 0.97.

### Motivation to Change Khat Use

The *Stages of Change Readiness and Treatment Eagerness Scale* [SOCRATES (42)] is an instrument that assesses the motivation to change substance use and is closely related to the transtheoretical model of behavior change (43). This theoretical approach was recently used in Ethiopia and has been found applicable in Ethiopian culture (44). The SOCRATES consists of 19 items (Likert scale from 1 “strongly disagree” to 5 “strongly agree,” and three subscales: Recognition (7 items; high scores mean respondents acknowledge problems with substance use, low scores that they deny problems with substance use), Ambivalence (4 items; high scores reflect more ambivalence, low scores more certainty; this subscale should be interpreted in relation to Recognition), and Taking Steps (8 items; high scores mean that respondents already started to change substance use behavior, low scores mean inactivity regarding change). The questionnaire was translated and adapted to assess motivation to change khat use for this study, and versions in Amharic and Afaan Oromoo were developed. We used the original wording and replaced the word “drug” with the word “khat” (sample item “*Sometimes I wonder if my khat is hurting other people*”). We found good to excellent Cronbach's alphas for two sub-scales in the three different languages: recognition Amharic (α = 0.80), Afaan Oromoo (α = 0.80), and English (α = 0.75); Taking Steps Amharic (α = 0.86), Afaan Oromoo (α = 0.82), and English (α = 0.86). The internal consistency coefficients of Ambivalence ranged between poor and acceptable, Amharic (α = 0.57), Afaan Oromoo (α = 0.60), and English (α = 0.48). This mirrors the results of Miller and Tonigan (42) who also found the Ambivalence subscale the least internally consistent. In the English version, the deletion of item 6 (Ambivalence subscale, “*Sometimes I wonder if my khat use is hurting other people*”) would have increased alpha to 0.750. Because of the small number of participants who used the English questionnaire (i.e., 2.3%, see below) and to retain comparability across all three languages, we used all items in the analyses. For the interpretation of the results, we use the guidelines provided by Miller and Tonigan (42) that are based on the results of the Project MATCH with alcohol users, for instance, we used the reported median scores to define groups of subjects with high and low values in each of the three subscales.

### Academic Performance

The participants' academic performance was assessed, asking for the cumulative grade point average (CGPA) of the previous study year as it has been frequently done by other studies (11).

### Ethics

The Institutional Review Boards of Jimma University (Ethiopia) and the University of Konstanz (Germany) approved this study. Informed consents were prepared, translated, and back-translated in Amharic and Afaan Oromoo, and participants were only included in the study after they had read, agreed, and signed the translated informed consent according to their language preferences. The study was part of a trial with the registry number NCT03730805.

### Statistical Procedure

SPSS 25 had been used to analyze the data. For statistical testing, two-tailed α = 0.05 was used. Outliers were identified by the procedure suggested by Hoaglin and Iglewicz (45): Any score greater than the upper quartile plus 1.5 times the interquartile range was identified as an outlier, and any score greater than the upper quartile plus 3 times the interquartile range was identified as an extreme case (46). Therefore, reporting consumption of more than 143 khat bundles in 28 days was defined as an extreme case and based on that, 15 participants were excluded from the study.

Because statistical pre-requirements of multiple imputation techniques were not given in all the self-report instruments (some individuals had left out a high number of items), we adopted the following procedure: all participants were excluded from data analysis who had left out more than two items in the same self-report instrument; for the remaining cases, missing items were replaced by the respective scale mean.

Differences between categories of participants were tested with one-way ANOVAs or *t*-tests (when variances were not equal: Mann-Whitney *U* tests). In addition, the relationship between khat use measures (days of khat use in last 28 days and bundles of khat used in the last 28 days) with psychopathology, trauma load, alcohol use, and motivation to change was computed with the non-parametric Spearman rank-order correlation coefficient because of deviations from normality (Kolmogorov-Smirnov and Shapiro-Wilk tests with *p* <.001).

Linear regressions were used to identify predictors of the two khat use variables (bundles of khat and days with khat use in the past 28 days). The assumptions for linear regression were tested: normality was checked using the Kolmogorov-Smirnov and Shapiro-Wilk tests and inspection of QQ-plots for khat bundles, AUDIT, SRQ, LEC, and PCL. The results show that normality cannot be assumed (*p* <0.001). Homoscedasticity was assured by visual inspection of scatter plots of residuals. We found no sign of multicollinearity (VIF < 2.16, Tolerance >.46). Cook's Distances revealed no highly influential cases (values ranged between 0.39 and 0.12). Because of the high number of participants and the method's robustness, we decided to use linear regressions despite violations against assumptions. In Step 1, we entered the independent variables age, CGPA, and AUDIT. In Step 2, we entered the sum scores of the SRQ-20, the PCL-5, and the LEC. In Step 3, we entered the three subscales of the SOCRATES. For each step, we calculated the goodness-of-fit for the overall model using the Akaike information criterion (AIC) and calculated *R*^2^ and adjusted *R*^2^. Finally, we used an automated stepwise procedure to remove non-significant predictors from the model using the F value (*p* > 0.10) to establish a final model.

## Results

### Descriptive Analysis

The average age of participants was 22.1 years (*SD* = 1.58; ranging between 17 and 28 years). The median reported study year was 4 (*M* = 3.46, *SD* = 1.05). The participants belonged to the following faculties, colleges, and departments: 362 (63.0%) participants were from the Institute of Technology, 64 (11.1%) from the Faculty of Natural Sciences, 58 (10.1%) from the College of Business & Economics, 45 (7.8%) from the Faculty of Medicine and Health, and 46 (8.0%) from Law, Social Science, and Humanities. Participants selected the following languages to fill the questionnaires: 365 (63.5%) Afaan Oromoo, 197 (34.3%) Amharic, and 13 (2.3%) English. Students described their monthly income as follows: 59 (10.3%) participants had < 100 Ethiopian Birr, 152 (26.4%) students indicated 100–300 Birr, 146 (25.4%) 301–500 Birr, 104 (18.1%) 501–700 Birr, 49 (8.5%) 701–900, and 46 (8.0) said they had more than 900 Birr; 19 (3.3%) participants were not willing to give information on monthly income. At the time of the study, 100 Birr was approximately $3.50. The mean cumulative grade point average was 3.01 (*SD* = 0.44, ranging between 1.63 and 3.89).

The sample consumed an average amount of 31.55 standard khat bundles (*SD* = 28.53) in the 28 days before the assessment; in this period, khat use was practiced on average on 15.11 days (*SD* = 8.54). Therefore, based on the criterion described above, 17.0% (*N* = 98) of the participants showed a highly problematic khat use.

Of the 575 participants, 124 (21.6%) reported that there was a time in their lives when they were unable to work, go to school, or fulfill household or childcare duties for at least 4 weeks due to mental or nerve-related problems; of them, 103 (17.9% of the total sample) reported the problem persisted in the last 4 weeks. Ever in their lives, 137 (23.8%) sought assistance from a counselor, medical doctor, priest, sheik, or healer because of mental or nerve-related problems; of them, 60 (10.4% of the total sample) sought such assistance in the last 4 weeks. Because they had no medical support yet, 64 participants (11.1%) were directly referred by our study team to the university's student clinic.

In the LEC, on average, 5.54 (*SD* = 4.56) types of potentially traumatic experiences were reported. In the PCL-5, a most severe traumatic event was reported by 343 participants. Of them, 135 reported car accidents, 91 sexual, physical, or emotional abuse, 39 deaths of close family members, 35 war and violence, 29 fire accidents, 7 indicated to have witnessed a peer suicide, and 7 experienced a natural disaster. Furthermore, 73 students reported that the stressful event happened directly to them, while 115 students reported that they had witnessed the situation, and in 89 cases, it had happened to close family members or friends. Seventeen students reported the stressful incidents had happened in the context of their profession (e.g., paramedic, military, police). Taken together, the PCL-5 sum score was on average 27.57 (*SD* = 19.88); 37.9% (*N* = 218) qualified for a positive screening result for PTSD.

The AUDIT sum score in the total sample reached a mean of 9.20 (*SD* = 10.11). Among all participants, 240 students (41.7%) reported no alcohol use in the last month; among the 335 students who used alcohol in the last month, the mean AUDIT sum score was 15.79 (*SD* = 8.45), which is in the range of medium risk alcohol use. Thus, scores above the cut-off of 8 (hazardous alcohol use) were reached by 47.1% (*N* = 271) of all respondents. Based on their scores, participants with hazardous alcohol use were grouped into the two severity categories as follows: 100 students (17.4% of all participants; *M* = 11.14, *SD* = 2.37) showed a medium-level and 171 a high-level alcohol problem (29.7% of all participants; *M* = 22.76; *SD* = 5.10).

The mean value of the SRQ-20 sum score (symptoms of Common Mental Disorders, CMD) was 9.81 (*SD* = 5.54), which is above the cut-off point of 8. In total, 60.2% (*N* = 346) of respondents screened positive for CMD. Of those who screened positive for CMD, 183 (i.e., 31.8% of the whole sample) also screened positive for PTSD, 189 (i.e., 32.8% of the whole sample) also screened positive for hazardous alcohol use, and 106 (i.e., 18.4% of the whole sample) also reported a lifetime functioning problem due to a mental disorder.

**Figure 1:**
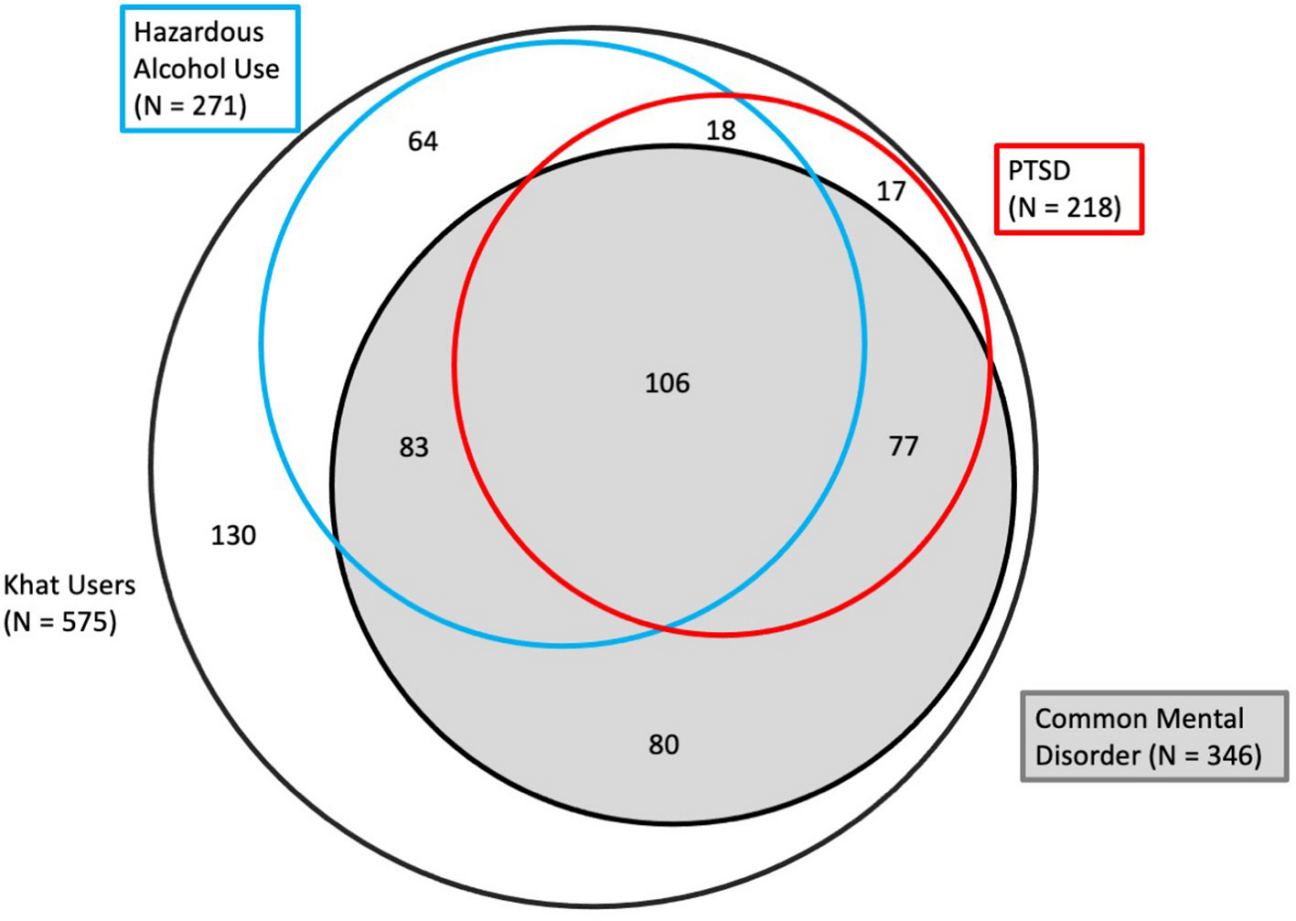
Venn Chart, displaying the overlap between groups of cases screening positive for Common Mental Disorders (CMD), PTSD and hazardous alcohol use among 575 students seeking for treatment because of their khat use (areas in the chart are approximately proportional to the size of the respective subsample).

The overlap of the three different bivariate categories of mental health problems among khat users (based on screening by SRQ-20, PCL-5, and AUDIT) in our sample is displayed in Figure 1. Only 22.6% (*N* = 130) did not score positively in any of these categories; 28.0% (*N* = 161) scored positively in one category, 30.9% (*N* = 178) in two, and 18.4% (*N* = 106) in three.

In the Stages of Change Readiness and Treatment Eagerness Scale (SOCRATES-khat), respondents' average score for Recognition was 24.88 [*SD* = 5.9; “very low” according to Miller and Tonigan (42)], the average score for Ambivalence was 13.74 (*SD* = 3.41; “low”), and the average score for Taking Steps was 26.94 (*SD* = 7.04; “low”). According to the original authors' guidelines and based on the US norms, 88.7, 54.3, and 76.5% of participants scored below the median (low magnitude of the motivational component) for the subscales Recognition, Ambivalence, and Taking Steps, respectively. The interpretation of the Ambivalence score is only possible in relation to Recognition, that is, 302 subjects scored low in both subscales (52.8% of the total group), and 10 subjects with low Ambivalence scored high in Recognition (i.e., 1.7%).

To estimate the effects of acute khat intoxication on self-report, we analyzed the association of the amount of recent khat intake on self-report data. There were no associations between the amount of khat used on the assessment day or the days before it and any clinical questionnaire outcomes. However, the SOCRATES-khat subscale *Taking Steps* showed significant but weak negative correlations with the amount of khat used on the days before the assessment (day of assessment: *r* = −0.094, *p* = 0.026; day before the assessment: *r* = −0.142, *p* = 0.001; 2 days before the assessment: *r* = −0.150, *p* < 0.001).

### Bivariate Associations Between Variables

Bivariate correlations between variables are reported in Table 1. Khat use variables showed weak to medium associations to measures of trauma and psychopathology as well as small associations to motivation to change.

**Table 1:** Bivariate Spearman rank-order correlations (*p*-values in parentheses) in 575 khat-using Jimma University students between days of khat use, khat use in bundles, trauma load (LEC sum score), PCL-5 sum score, SRQ-20 sum score, AUDIT sum score, and SOCRATES subscales.

	**Khat use in bundles (last 28 days)**	**LEC**	**PCL-5**	**SRQ-20**	**AUDIT**	**SOCRATES Recognition**	**SOCRATES Ambivalence**	**SOCRATES Taking Steps**
Days with khat use in the last 28 days	0.831 (<0.001)	0.066 (0.117)	0.006 (0.115)	0.102 (0.014)	−0.005 (0.913)	0.095 (0.023)	0.042 (0.315)	−0.125 (0.003)
Khat use in bundles in the last 28 days		0.125 (0.003)	0.124 (0.003)	0.112 (0.007)	0.042 (0.317)	0.112 (0.007)	0.065 (0.120)	−0.086 (0.040)
LEC-5			0.523 (<0.001)	0.360 (<0.001)	0.200 (<0.001)	0.199 (<0.001)	0.162 (<0.001)	0.101 (0.015)
PCL-5				0.557 (<0.001)	0.225 (<0.001)	0.348 (<0.001)	0.311 (<0.001)	0.199 (<0.001)
SRQ-20					0.271 (<0.001)	0.396 (<0.001)	0.343 (<0.001)	0.154 (<0.001)
AUDIT						0.121 (0.004)	0.111 (0.008)	0.067 (0.108)
SOCRATES Recognition							0.637 (<0.001)	0.513 (<0.001)
SOCRATES Ambivalence								0.562 (<0.001)

*SRQ-20, Self-Report Questionnaire 20; LEC-5, Life-Event Checklist for DSM-5; PCL-5, PTSD Checklist for DSM-5; AUDIT, Alcohol Use Disorder Identification Test; SOCRATES, Stages of Change and Treatment Eagerness Scale*.

Using, on average, two or more bundles per day, that is, problematic khat use, was associated with more reported traumatic experiences (LEC, *M* = 5.7, *SD* = 4.5 vs. *M* = 6.8, *SD* 4.9, *t* = 2.276, *p* = 0.023) and there were trends toward more PTSD symptoms (PCL-5: *M* = 26.9, *SD* = 19.5 vs. *M* = 31.1, *SD* = 21.5, *t* = 1.922, *p* = 0.055) but no difference in alcohol problems and common mental disorders (AUDIT: *M* = 8.9, *SD* = 9.9 vs. *M* = 10.9, *SD* = 10.1, *z* = 1.469, *p* =.142; SRQ-20: *M* = 9.7, *SD* = 5.4 vs. *M* = 9.9, *SD* = 6.0, *z* = 0.388, *p* =.698). The SOCRATES subscales showed no association to problematic khat use (−.042 ≤ *r*s ≤.08; *p*s ≥.057).

SOCRATES subscales showed small correlations to khat use and alcohol measures but medium level associations to the clinical scales; thus, the higher the clinical burden, the higher the respective SOCRATES subscale.

### Linear Regression to Predict Khat Use

Hierarchical regression analyses were used to determine predictors of the khat use variables *amount of khat used in the last 28 days* and *days with khat use in the last 28 days* (see Tables 2, 3). For days of khat use, variables of Step 1 accounted for 1.3% of the variance (overall model test: *F*_3,571)_ = 2.411, *p* = 0.066). The mental health variables of Step 2 accounted for an additional 1.1% of the variance (overall model test: *F*_6,568)_ = 2.283, *p* = 0.035). In the third step, motivation to change variables (Recognition, Ambivalence, and Taking Steps) were added and the model accounted for an additional 4.0% of the variance (overall model test: *F*_9,665)_ = 4.325, *p* < 0.001). After backward stepwise deletion of insignificant predictors, the final model contained the predictors age, Recognition, and Taking Steps and explained 5.8 % of the variance (overall model test: *F*_3,571)_ = 11.639, *p* < 0.001).

**Table 2:** Summary of the linear regression model of dependent variable days of khat use in the last 28 days.

	**Step 1**				**Step 2**				**Step 3**				**Final model**			
**Predictors**	**B**	**SE B**	**ß**	***P***	**B**	**SE B**	**ß**	***P***	**B**	**SE B**	**ß**	***P***	**B**	**SE B**	**ß**	***P***
Constant	25.870	5.637		<0.001	24.694	5.652		<0.001	26.465	5.715		<0.001	27.287	5.017		<0.001
Age	−0.558	0.226	−0.103	0.014	−0.568	0.225	−0.105	0.012	−0.578	0.222	−0.107	0.009	−0.567	0.220	−0.105	0.010
CGPA	0.392	0.809	0.020	0.628	0.347	0.807	0.018	0.667	0.172	0.793	0.009	0.828	-	-	-	-
AUDIT	0.043	0.036	0.051	0.230	0.010	0.038	0.012	0.795	0.005	0.037	0.005	0.901	-	-	-	-
SRQ-20	-	-	-	-	0.109	0.080	0.071	0.170	0.048	0.081	0.031	0.553	-	-	-	-
PCL-5	-	-	-	-	0.010	0.023	0.022	0.680	0.007	0.023	0.015	0.775	-	-	-	-
LEC-5	-	-	-	-	0.082	0.091	0.044	0.364	0.086	0.089	0.046	0.335	-	-	-	-
Recognition	-	-	-	-	-	-	-	-	0.245	0.086	0.171	0.004	0.326	0.070	0.227	<0.001
Ambivalence	-	-	-	-	-	-	-	-	0.126	0.149	0.050	0.397	-	-	-	-
Taking Steps	-	-	-	-	-	-	-	-	−0.302	0.064	−0.249	<0.001	−0.288	0.059	−0.237	<0.001
F	*F*_3,571_ = 2.411, *p* = 0.066				*F*_6,568_ = 2.283, *p* = 0.035				*F*_9,565_ = 4.325, *p* < 0.001				*F*_3,571_ = 11.639, *p* < 0.001			
AIC	2,466.629				2,466.163				2,447.555				2,439.738			
*R* ^2^	0.013				0.024				0.064				0.058			
Adj. R^2^	0.007				0.013				0.050				0.053			
*F* Change	*F*_3,571_ = 2.411, *p* = 0.066				*F*_3,568_ = 2.141, *p* = 0.094				*F*_3,565_ = 8.235, *p* < 0.001				-			

*CGPA, cumulative grade point average; AUDIT, Alcohol Use Disorder Identification Test; SRQ-20, Self-Report Questionnaire 20; PCL-5, PTSD Checklist for DSM-5; LEC-5, Life-Event Checklist for DSM-5; AIC, Akaike information criterion*.

**Table 3:** Summary of the linear regression model of dependent variable bundles of khat use in the last 28 days.

	**Step 1**				**Step 2**				**Step 3**				**Final model**			
**Predictors**	**B**	**SE B**	**ß**	***P***	**B**	**SE B**	**ß**	***P***	**B**	**SE B**	**ß**	***P***	**B**	**SE B**	**ß**	***P***
Constant	55.153	18.849		0.004	53.464	18.884		0.005	57.024	19.248		0.003	59.007	16.911		0.001
Age	−1.333	0.755	−0.074	0.078	−1.426	0.753	−0.079	0.059	−1.443	0.747	−0.080	0.054	−0.1381	0.742	−0.077	0.063
CGPA	1.307	2.706	0.020	0.629	1.120	2.697	0.017	0.678	0.691	2.672	0.011	0.796	-	-	-	-
AUDIT	0.208	0.119	0.074	0.082	0.108	0.127	0.038	0.392	0.096	0.125	0.034	0.445	-	-	-	-
SRQ-20	-	-	-	-	0.002	0.266	0.000	0.993	−0.171	0.274	−0.033	0.533	-	-	-	-
PCL-5	-	-	-	-	0.098	0.078	0.068	0.208	0.088	0.079	0.061	0.264	-	-	-	-
LEC-5	-	-	-	-	0.425	0.303	0.068	0.161	0.443	0.300	0.071	0.140	0.601	0.263	0.096	0.023
Recognition	-	-	-	-	-	-	-	-	0.531	0.288	0.111	0.066	0.762	0.241	0.159	0.002
Ambivalence	-	-	-	-	-	-	-	-	0.662	0.501	0.079	0.187	-	-	-	-
Taking Steps	-	-	-	-	-	-	-	-	−0.824	−216	−0.203	<0.001	−0.721	0.200	−0.178	<0.001
*F*	*F*_3,571_ = 1.904, *p* = 0.128				*F*_6,568_ = 2.187, *p* = 0.043				*F*_9,565_ = 3.203, *p* = 0.001				*F*_4,570_ = 6.235, *p* < 0.001			
AIC	3,853.894				3,853.481				3,844.000				3,837.993			
*R* ^2^	0.010				0.023				0.049				0.042			
Adj. R^2^	0.005				0.012				0.033				0.035			
*F* Change	*F*_3,571_ = 1.904, *p* = 0.128				*F*_3,568_ = 2.457, *p* = 0.062				*F*_3,565_ = 5.140, *p* = 0.002				-			

*CGPA, cumulative grade point average; AUDIT, Alcohol Use Disorder Identification Test; SRQ-20, Self-Report Questionnaire 20; PCL-5, PTSD Checklist for DSM-5; LEC-5, Life-Event Checklist for DSM-5; AIC, Akaike information criterion*.

For bundles of khat use, variables of Step 1 accounted for 1.0% of the variance (overall model test: *F*_3,571)_ = 1.904, *p* = 0.128). The mental health variables of Step 2 accounted for an additional 1.3% of the variance (overall model test: *F*_6,568)_ = 2.187, *p* =0.043). In the third step, motivation to change (Recognition, Ambivalence and Taking Steps) accounted for an additional 2.6% of the variance (overall model test: *F*_9,565)_ = 3.203, *p* =0.001). After backward stepwise deletion of insignificant predictors, the final model contained the predictors age, LEC-5 (trauma load), Recognition, and Taking Steps (overall model test: *F*_4,570)_ = 6.235, *p* < 0.001; *R*^2^ = 0.042).

## Discussion

In this study, we report on the characteristics of a khat-using student sample of an Ethiopian university in a traditional khat growing region who seek psychological assistance to reduce or stop khat use. We studied the relationship of quantitative khat use variables to mental health variables and motivation to change. The sample reported a very high khat use, on average, more than 30 bundles in 4 weeks and use on more than every other day. A highly problematic amount of use as pragmatically defined based on previous research was evident in about 17.0% of the sample. Although half of the sample used khat on the day of the assessment, we found no effect on the self-report of clinical symptoms.

Furthermore, the sample reported a high trauma load and a high mental health burden; 17.5% reported current severe functioning problems due to mental health problems, 60.2% screened positive for CMD, 37.9% for PTSD, and 47.1% for hazardous alcohol use. Almost half fulfilled two or more of these mental health criteria in addition to khat use. According to the norms based on alcohol users in the US, the motivation to change khat use as measured by the SOCRATES was generally low. On this background, the very low Recognition score can be interpreted that subjects did not admit to having problems concerning their khat use. In this context, the low Ambivalence score means that the majority of subjects have little ambivalence regarding their khat use because they do not see problems caused by khat use, whereas the low scores on Taking Steps can be interpreted in a way that subjects are not already doing something to prepare a change of their khat use. However, we found a weak association of khat use just before the assessment on this subscale; that is, the more khat was used, the lower the readiness to prepare for a change. The bivariate correlations revealed only weak associations between the 28-days khat use variables, mental health measures, and motivation to change. In the statistical prediction of khat use, the regression models were only able to explain small percentages of the variance of the two use variables (<6%); the strongest associations were found between khat use and motivation to change.

This study substantiates the conclusion of the recently published meta-analysis (9) that khat use is a severe problem among Ethiopian University students; in our treatment-seeking sample, we were able to recruit about 1.4% of all students at JU and found extremely high self-reports of khat use. Our findings also correspond to the recent meta-analysis by Mekuriaw et al. (22), who observed that students using khat are often also affected by common mental disorders; we found a similar CMD prevalence rate in our study and were able to report in more detail the quality of mental health problems. The high rate of participants screening positive for PTSD might be related to the specific population exposed to violence in the context of political protests in the years before the assessment. Our data support the commonly employed method by using the SRQ-20 to detect CMD cases because almost all cases with positive PTSD screening on the PCL-5 (183 of 218, i.e., 83.9%) and with functioning problems due to mental disorders (106 of 124, i.e., 85.5%) scored above the respective cut off (7/8). Our results add further information on the high burden of khat-using university students by showing their hazardous use of alcohol and their severe functioning problems. Our data correspond to the findings of other studies: for instance, Teklie et al. (25) found the proportion of male khat users who used alcohol after chewing khat being more than 40% in the age group of 15–29 years; the meta-analysis of Ethiopian student samples by Gebrie et al. (9) found alcohol use was strongly associated with khat use. Our findings also show that most of these psychologically high burdened khat users do not regard khat use as their main problem, neither worrying about it nor wanting to change it. Our hypotheses can only be partially confirmed because of the smaller than expected (statistically significant but negligible) associations between khat use and mental health respective motivation to change shown in bivariate associations and multiple regression models. Our data also show that like among young adults users from western countries substance use among JU students was related inversely to indicators of insight and risk perception (47)—the contrary was found for subjects with already established alcohol use disorder (48).

The most obvious question arising from our data is why do subjects with little motivation to change their khat use participate in this study? Three lines of arguments might attempt to explain our findings. First, both the SOCRATES as an assessment tool and the related transtheoretical model (TTM) of change have rarely been used in the Ethiopian context. Using a totally different method to assess motivation to change khat use in the context of TTM, Estifanos et al. (49) found a different picture: they used the respondents' intention to quit khat use and found that nearly half of the sample wanted to stop khat use within the next 6 months (i.e., contemplation stage) and 14% within the next 30 days (i.e., preparation stage). Also, some other studies assessed the wish to stop khat use with a single item [e.g., (50)]. The method used in this study differs clearly because a 19-item instrument was used that measured a much more complex concept of motivation to change. It seems likely that SOCRATES-khat items like “*Sometimes I wonder if I am a khat addict*” provoked reactance because khat addiction is highly stigmatized. A different bias—social desirability—might be especially relevant in the study by Adugna et al. (50). Adugna and colleagues conducted their study in a part of the country where khat use is not a tradition and where the dominant Orthodox church condemns the substance publicly—in contrast to our study site, where khat use is a tradition and most people are Muslims whose religion accepts the substance. Although we developed the Amharic and Afaan Oromoo versions of the instrument with care, the English language version produced similar results, and the high intercorrelation of subscales was reproduced in the Ethiopian sample, more empirical evidence is needed to interpret the answers in the cultural and societal context. The general societal attitude toward khat might be fundamentally different from the attitudes of Western societies toward alcohol and, thus, a country-specific norm is needed to interpret the data assessed with this instrument. Second, participants in this study might have mainly sought assistance for their comorbid mental health problems when enrolling in the study because they might not have been aware of other ways to receive assistance; khat might in this context be a functionally used substance, for instance, to modify mood states or to self-medicate symptoms (51). Beyond the pharmacological effects, the social support of a khat-using group might be an important resource for students with mental health problems; the colloquial term “Jezbas” denotes a segregated group of students who use khat or other substances together. The influence of such informal support networks of substance users has been linked to lower motivation for abstinence (52). This argument, thus, states that khat in the context of missing formal assistance might be perceived as something helpful to cope with one's mental problems instead of a problem itself. Third, participants might have enrolled in the study because they expected other benefits. However, this is unlikely because all participants were informed beforehand about the (small) compensation for public transport and missed meals. In sum, it is most likely that the American norms that we had used to interpret the results were not adequate, that is, have been too high. Assuming that the direction of association is the same as in alcohol studies, the magnitude of khat-related answers in Ethiopia is lower than alcohol-related answers in the US; the hierarchical regression analysis can, thus, be interpreted as it is presented below.

The results of the hierarchical regression analysis revealed a low proportion of explained variance as well as small regression coefficients and bivariate correlations between previously identified predictors of khat use and quantitative measures of consumption. This is probably due to the characteristics of this study involving a more homogeneous treatment-seeking sample compared to the previously published studies with representative samples. In addition, our statistical models revealed a positive association of motivation to change to the khat use measures when controlling for other variables. This is a typical result also found for other substances [e.g., (48)] and that is often explained in the context of health behavior theories: people practicing a risk behavior (e.g., substance abuse) evaluate their subjective risk for negative consequences higher than control subjects who do not engage in it and, in turn, are more motivated to engage in precautionary behaviors or abandoning the risk behavior [e.g., (47)]. Recent studies showed that khat-using university students experience clear symptoms of an addiction syndrome (10) and often experience failed attempts to stop khat use (53). Together with these results, our data underscore that it is highly needed and promising to offer specialized prevention and clinical support services for students with khat-use problems.

A striking finding is the low participation of female students in this voluntary convenience sample. Even though khat use is also practiced among female students in the study region [Alemseged et al. (54): 16% of females used khat in the general population; Dires et al. (12): 29% females among khat using high school students; Abdeta et al. (10): 15% of female undergraduate university students], a tiny proportion of female students sought assistance because of their use of this substance in this study (i.e., only 9 of the initial 717 students, 1.3%). Although the reasons for this mismatch need to be clarified in future studies, we do not think that recruitment information did not reach female students because printed flyers and announcements were publicly available. We hypothesize that the small proportion of women who volunteered for this study is related to the subjective fear of discrimination because, in large parts of Ethiopia, female khat use is seen as unacceptable (14) and has been strongly linked to female moral decay (55). It remains unclear whether this effect is typical for our study site or can be generalized to the whole of the country. If this is true, it will need special strategies to engage female khat users in future counseling and clinical services for this substance. In this sense, Kerebih et al. (28) found that respondents with CMD were less frequently looking for mental health assistance when they used khat.

## Strengths and Limitations of the Study

The study used instruments that had been employed in different continents and cultures; for some of them, Ahmaric and Afaan Oromoo versions were developed for this study. There had been no or few prior validation studies in Ethiopia, so country-specific norms for interpretation of the scores are missing, and there is little experience with the use of these versions of the instruments in general. While we are still confident that the Amharic and Afaan Oromoo versions of the instruments were at least as valid as English versions would have been, this should be considered when interpreting the results.

Using electronic devices for research in Ethiopia is new and has been practiced only by a few studies [e.g., (53)], so that no information is available on potential biases related to the use of this technique in Ethiopia. Using electronic devices to assess sensitive information without an interviewer or counselor being present has the potential to overcome answer biases (such as social desirability) and reach larger numbers of participants. However, it also might create mistrust in countries where the government strongly controls the internet. Therefore, the electronic assessment methods need to be tested more in the Ethiopian context. In other countries, electronic substance use assessment and intervention programs were highly accepted by students [e.g., (56)].

We studied a treatment-seeking sample of khat users using a convenience sampling method. Preferably, treatment seekers should have been sampled using random sampling methods because otherwise, the generalizability of the results is unclear. However, the employed method allowed typical conclusions such as the difficulty of enrolling females. In general, there are no studies with treatment-seeking samples of student khat users, and this study can be seen as a pilot study that needs to be replicated; only one other treatment study also using convenience sampling identified a group of khat-users among refugees in Nairobi that were equally burdened with mental health problems (17). A replication study should also include a representative sample from all universities and more sophisticated measures to fully assure that participants' recent substance use will not affect testing results.

## Conclusion

This study highlights the challenges of studying substance use and the need for planning interventional strategies for khat as well as for comorbid mental health problems among university students in the countries of the khat belt. Instruments and national norms need to be developed to effectively screen for individuals in need of professional support and measure change. Regarding prevention and intervention, our study clearly shows how urgently adequate professional assistance services need to be strengthened at Ethiopian universities and that the to-be-built-up services need to be prepared for clients with high and complex mental health needs. The high number of participants who needed to be directly referred to clinical services shows that typical substance counseling services will largely be overburdened. We expect that khat counseling alone will not be sufficient to serve the needs of this group. Furthermore, the data suggest that there are barriers to the utilization of counseling services for female khat users. Therefore, research on effective and efficient intervention and prevention strategies for this group of khat users and research on the methods of their implementation at Ethiopian universities is required.

## Data Availability Statement

The raw data supporting the conclusions of this article will be made available by the authors, without undue reservation.

## Ethics Statement

The studies involving human participants were reviewed and approved by Konstanz University Ethics Commission, Box 214, 78457 Konstanz, contact Mrs Sabine Schieß, sabine.schiess@uni-konstanz.de, and Jimma University Institutional Review Board, Institute of Health, Jimma University, contact Prof. Dr. Zeleke Mekonnen, zeleke.mekonnen@ju.edu.et. The patients/participants provided their written informed consent to participate in this study.

## Author Contributions

MH contributed to the conception, design, data acquisition and analysis, interpretation, and drafted the manuscript. MO contributed to conception, design, data acquisition and analysis, interpretation, drafted and revised the manuscript. MS contributed to conception, design, data acquisition and analysis, interpretation, and revised the manuscript. LK and KA contributed to the conception, design, data acquisition and analysis, and revised the manuscript. MW, AZ, NB, EB, YY, SS, and JS contributed to the design, data acquisition, and revision of the manuscript. All authors approved the submitted version of the manuscript.

## Conflict of Interest

The authors declare that the research was conducted in the absence of any commercial or financial relationships that could be construed as a potential conflict of interest. The reviewer AA declared a shared affiliation, with one of the authors with two of the authors to the handling editor at the time of the review.

## Publisher's Note

All claims expressed in this article are solely those of the authors and do not necessarily represent those of their affiliated organizations, or those of the publisher, the editors and the reviewers. Any product that may be evaluated in this article, or claim that may be made by its manufacturer, is not guaranteed or endorsed by the publisher.
